# Prognostic Value of TGF-β Expression in Bladder Cancer: A Systematic Review and Meta-analysis

**DOI:** 10.5152/tud.2024.24024

**Published:** 2024-05-01

**Authors:** Shima Kianmehr, Mohammad Vahabirad, Atefeh Seghatoleslam, Erfan Sadeghi, Roozbeh Kiani, Hadi Ghasemi

**Affiliations:** 1 Department of Clinical Biochemistry, Hamadan University of Medical Sciences School of Medicine, Hamadan, Iran; 2 Department of Biochemistry, Shiraz University of Medical Sciences School of Medicine, Shiraz, Iran; 3 Department of Biostatistics, Shiraz University of Medical Sciences School of Medicine, Shiraz, Iran

**Keywords:** Bladder cancer, meta-analysis, prognosis, TGF-β

## Abstract

**Objective:**

Transforming growth factor beta (TGF-β) is a member of the growth factor superfamily that clinical studies address its association with bladder cancer invasion, progression, and metastasis. The present systematic review and meta-analysis aimed to explore the prognostic significance of TGF-β expression in bladder cancer patients.

**Materials and Methods:**

The major international databases, including PubMed, Web of Science, Embase, and Scopus, were searched for full-text literature citations. The hazard ratio (HR) with a 95% CI as the effect size was applied as the appropriate summarized statistic. We used a random-effects model using the DerSimonian and Laird method to estimate the pooled effect size. To assess the heterogeneity among trials, the I-square (*I* ^2^) statistic and Cochran’s Q test were used. Forest and funnel plots were drawn to respectively demonstrate the findings and detect any existing publication bias.

**Results:**

This meta-analysis included 3 studies that met the criteria and included 535 patients. The combined HR for the selected studies was 2.250 (95% CI = (1.411, 3.586), *P* < .001) and no significant heterogeneity was detected between trials (*I* ^2^ = 58.63, *P* = .089). Furthermore, no severe asymmetry was seen within the funnel plot, indicating a lack of potential publication bias.

**Conclusion:**

Our findings suggest that TGF-β expression can remarkably predict a worse prognosis in patients with bladder cancer. The results of the present meta-analysis may be validated through further updated reviews and additional relevant investigations in future studies.

**Main Points**

- A total of 535 participants from 3 trials were included in this meta-analysis.
- No significant heterogeneity was detected between trials.
- High expression of TGF-β could be a significant marker in predicting bladder cancer.

## Introduction

Bladder cancer is a prominent contributor to cancer-related mortality on a global scale.^1^ It ranks as the second most prevalent malignancy of the urinary tract worldwide and the tenth most prevalent cancer globally.^2^ The classification of bladder cancer is based on the degree of tumor infiltration into the bladder wall, resulting in 2 primary groups. The first one is non-muscular invasive bladder cancer (NMIBC), which occurs in the inner lining of the bladder, and the second one is muscle-invasive bladder cancer (MIBC), which penetrates the deeper layers of the bladder wall and may also involve nearby lymph nodes and other organs.^3,4^ Non-muscular invasive bladder cancers are the predominant form of bladder cancer, representing around 70% of all diagnosed cases. The remaining 30% of bladder cancers are classified as MIBCs.^5,6^ Small cell carcinoma, adenocarcinoma, and squamous cell carcinoma are the other less prevalent forms of bladder cancer.^5,7^

Non-muscular invasive bladder cancer patients have a 5-year survival rate of >90% but a recurrence risk of over 50%. Moreover, most patients undergo multiple therapies and cystoscopic surveillance, which lowers their quality of life^8,9^ This highlights the need to investigate novel indicators for predicting the outcome of cancer, selecting appropriate treatments, and managing patients. In this context, there are recently identified markers in the body fluids of humans that are pivotal in both the prognosis and diagnosis of cancer. For example, tumorigenic nucleic acids present in body fluids have the potential to function as noninvasive biomarkers, which would be especially beneficial in the context of early cancer detection, patient follow-up, and individualized treatment.^8^ According to a study by Su et al, circRIP2 promotes the progression of bladder cancer via the Tgf-2/smad3 signaling pathway.^10^

Transforming growth factor beta, a member of the superfamily of cytokines, has been shown to play multiple roles in physiopathological processes, including organ formation, embryonic development, tissue repair, homeostasis, tumor invasion, immune escape, metastasis, and therapeutic resistance.^11^ Owing to the role of TGF-β signaling in cancer development, any carcinogenic factors that activate TGF-β signaling can potentially induce tumor initiation, tumor metastasis and colonization, immune escape, and resistance to therapeutic medicine through various ways, including tumor cell epithelial-mesenchymal transition (EMT) induction, transformation of normal fibroblasts (NFs) into Cancer-associated fibroblasts (CAFs), and extracellular matrix (ECM) reconstruction.^12,13^ Lately, TGF-β has gained attention for its metabolic effect. In this regard, researchers have demonstrated that TGF-β can be a host and tumor metabolic reprogramming cytokine.^14,15^ Secretion of TGF-β by many tumor cells is shown to be associated with tumor growth and development as well as cancer immunity. During the canonical TGF-β signaling pathway, SMAD (Suppressor of Mothers Against Decapentaplegic) can control tumor growth, metastasis, and immune regulation.^16^ Intense clinical investigations revealed that TGF-β is overexpressed in various cancers, including malignant melanoma, bladder,^17^ colon,^18^ breast,^19^ esophagus,^20^ liver,^21^ stomach,^22^ lung,^23^ pancreas,^24^ kidney,^25^ and brain.^26^ Recent evidence has demonstrated that the TGF-β gene and protein are significantly overexpressed in bladder cancer^27^ and it is associated with invasive tumor stage, high grade, increased risk of tumor progression, and death from bladder carcinoma.^28^ As mentioned earlier, tumor cells up-regulate TGF-β expression to stimulate EMT, angiogenesis, and immunosuppression.^29^ Epithelial-mesenchymal transition is a crucial mechanism underlying bladder cancer invasion and metastasis.^12^ In addition to the essential role of the EMT process in the epithelial cancer cells acquiring migratory and invasive capabilities, the crosstalk with the tumor microenvironment (TME), including cytokines, inflammatory mediators, stromal cells, immune cells, and ECM has been shown to play an emerging role in cancer cell invasion and stemness.^30,31^ The poor prognosis and challenging issues in managing patients with bladder cancer and their treatment are mainly due to the local invasion and distant metastasis. Considering the significance of this marker in cancer, measuring TGF-β appears advantageous in advancing treatment objectives, prognosis, and management of bladder cancer.

Current bladder cancer diagnosis involves cystoscopy, urine cytology, and laboratory and instrumental studies. Cystoscopy is an invasive procedure, and void urine cytology has a low sensitivity; therefore, it is evident that a more dependable marker with greater prognostic value must be developed to enhance cancer treatment and management. Therefore, this systematic review and meta-analysis seek to determine whether TGF-β can be utilized as a prognostic indicator for bladder cancer.

## Materials and Methods

### Searching Strategy

PubMed, Scopus, Embase, and Web of Science were systematically searched from inception to 2023. The keyword terms used included: (i) “TGF-β” or “TGF-beta”; (ii) “Urinary bladder neoplasms” or “bladder cancer” or “bladder tumor”; (iii) “Prognoses” or “prognosis” or “prognostic factors” or “prognostic value.” Two independent investigators performed a manual search of the bibliographies of all relevant articles. Additionally, the papers were evaluated carefully to avoid duplication of records.

### Inclusion and Exclusion Criteria

The articles containing any of the following were included: TGF-β expression in bladder cancer as the main feature of the study, adequate data to assess HR, and case-control studies in overall survival. Exclusion criteria were as follows: duplicate data, review articles, unrelated or no available data, non-human models, papers without HR, and CI.

### Data Extraction and Statistical Analysis

The first author’s name, year of publication, number of cases, mean age of cases age, follow-up period, and HR were recorded for each study. Also, the hazard ratio (HR), as the effect size, with the corresponding 95% CI, was extracted for each trial. Two observers independently extracted data of interest. A random-effects model using the DerSimonian and Laird method was performed to estimate the pooled effect size. To assess the heterogeneity among trials, *I* ^2^ statistic and Cochran’s Q test were used. Forest and funnel plots were drawn to respectively demonstrate the findings and detect any existing publication bias. All analyses were done using Stata software (StataCorp. 2021. Stata Statistical Software: Release 17. College Station, TX: StataCorp LLC.)

## Results

### The Selection of Included Studies

**Figure 1.**
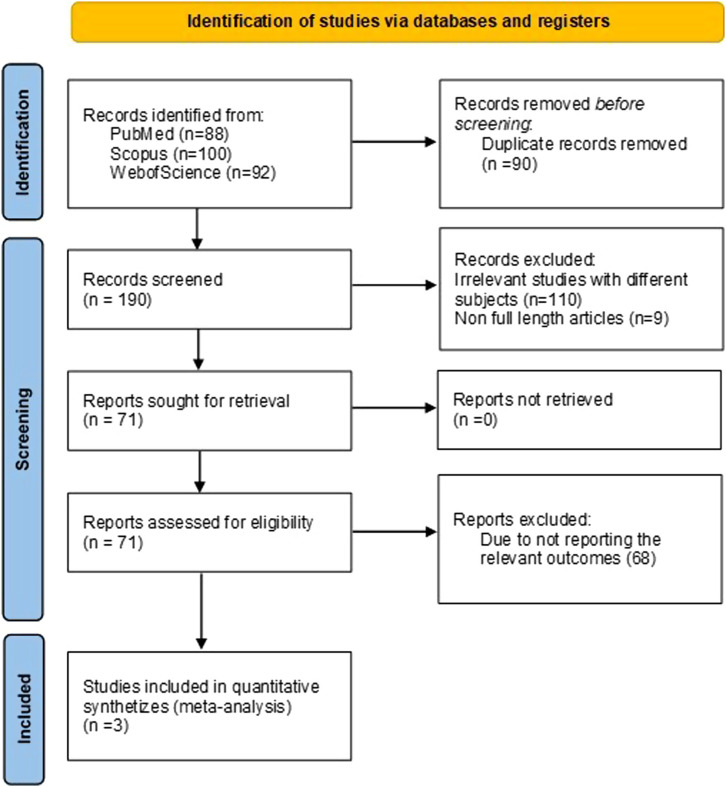
Prisma flowchart.

Our search strategy identified 280 studies. Of these, 90 duplicate articles were excluded by using EndNote software. Afterward, titles and abstracts were manually screened, during which 119 irrelevant papers were removed, and the remaining ones were selected to review full texts. Among them, 68 articles were excluded because they were case reports or were not eligible for inclusion in this study. Three articles published from their inception until August 2023 were ultimately included in the present study^15,32,33^ (Figure 1).

### Study Characteristics

The main features of the 3 studies included in our meta-analysis are presented in Table 1. The number of patients was a total of 535, comprising 424 men and 111 females. Of these 3 studies, 2 were conducted in the USA and 1 in Serbia. Immunohistochemical analysis and ELISA were used as identical methods in these studies. In the included studies, the TGF-β1 immunoexpression was deemed high when at least 50% of cancer cells showed moderate or strong color intensity in immunohistochemistry. Additionally, the cut-off value for plasma TGF-β levels of healthy males in ELISA was 5 ng/mL in records using the ELISA method. Patients in Stojnev, S. et al's study received TURBT ± mitomycin, Intravesical BCG, Cystectomy, and Chemo/radiotherapy, although the 2 other studies treated patients by radical cystectomy and pelvic lymphadenectomy. The combined duration of follow-up for these studies varied between 25 and 125 months.

### Overall Survival Meta-Analysis Results

**Figure 2.**
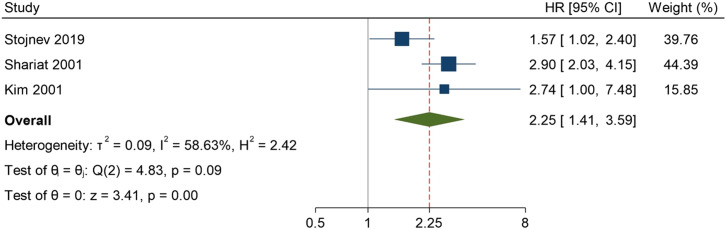
Forrest plot presenting results of the random-effects model and the estimated pooled hazard ratio for overall survival. HR, Hazard ratio.

**Figure 3.**
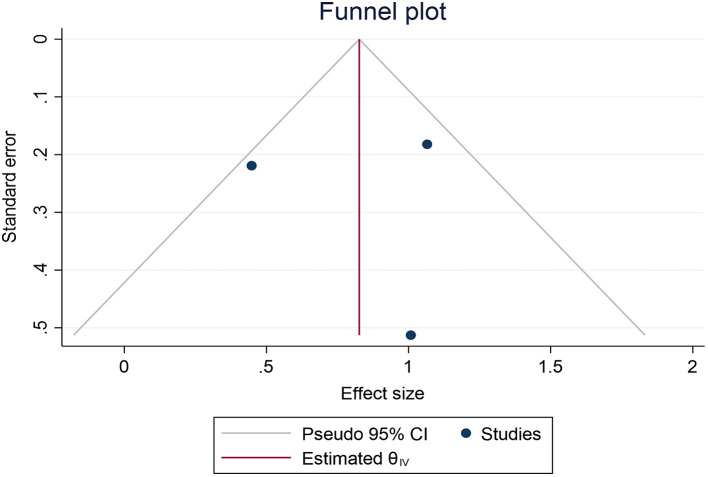
Funnel plot for assessment of publication bias.

A total of 535 participants from 3 trials were included in the analysis. The model revealed that high expression of TGF-β was associated with a 2.25 times higher risk of mortality compared to low expression (HR = 2.250, 95% CI = (1.411, 3.586), *P* < .001). Moreover, no significant heterogeneity was detected between trials (*I* ^2^ = 58.63, *P* = .089) (Figure 2). Furthermore, no severe asymmetry was seen within the funnel plot, indicating no potential publication bias (Figure 3).

## Discussion

As far as we are aware, this meta-analysis is the first to assess the TGF-β expression prognostic value in patients with bladder cancer, although only 3 records have reached the inclusion criteria for this meta-analysis. Several studies have reported that TGF-β expression is correlated with tumor growth and invasion as well as survival in patients with bladder cancer.^34,35^ Furthermore, it was observed that higher expression of TGF-β is associated with more advanced tumor stages, lymph node metastasis, and lymphovascular invasion in patients.^32^ Provided information approved that TGF-β may play a crucial role in the formation of matrix and favorable conditions for occurrence and development of cancer.^36-38^ In this regard, it is well known that TGF-β, as a growth factor, plays a key role in the regulation of extracellular matrix composition in cancers, particularly in bladder cancer.^39,40^ Owing to the pro-oncogenic activity of TGF-β1, it can regulate the nuclear localization of the smad2/3-smad4 complex as the main inducer of epithelial-mesenchymal transition (EMT) in bladder cancer. During tumorigenesis, the induction of the EMT process leads to a progressive loss of polarity and adhesions of normal cells and gain of invasive and migratory ability, as well as production of extracellular matrix components.^41,42^ During EMT, cancerous cells lose cell-cell adhesion junctions and eventually increase cancer stemness, resulting in higher mobility and invasiveness.^43^ The nuclear localization of Hippo pathway transcriptional effectors, Yes-associated protein (YAP), and TAZ (WW domain containing transcription regulator 1, or WWTR1) have also been described to be actively regulated with TGF-β1 related signaling pathway.^33,44^ In this regard, the cross-talk between the Hippo pathway and TGF-β1 has been addressed in previous studies.^45^ Moreover, epidemiological studies using clinical data also suggest that elevated TGF-β1 expression may contribute to the progression of NMIBC to MIBC, leading to a worse prognosis.^35^ The prognostic value of TGF-β in bladder cancer was evaluated using meta-analysis in the present study. According to our findings, the elevated expression of TGF-β was correlated with mortality and decreased survival in patients with bladder cancer, and it could be concluded that TGF-β might be a potential prognostic marker for patients who suffer from bladder cancer.

While our results are promising, there are some constraints in our meta-analysis. Although the total number of studies enrolled through the initial search of databases was 280 records, the studies that took part in the final quantitative data extraction were 3, and therefore the number of records included was relatively small. Another limitation of our study is that using different antibodies in IHC and ELISA methods in the included studies may give rise to bias and a higher amount of heterogeneity at the same time. Besides, we could not account for potential confounders, such as differences in treatment protocols, patient characteristics, and comorbid conditions across studies. Therefore, further updated meta-analyses with a larger number of included studies are necessary to validate the present findings in the future.

Based on the findings of the current systematic review and meta-analysis, it is apparent that elevated levels of TGF-β expression may serve as a substantial prognostic indicator for patients diagnosed with bladder cancer. It is possible, however, that the restricted quantity of investigations incorporated in our meta-analysis compromises the reliability of this finding. Thus, these observations must be corroborated and verified through subsequent pertinent investigations employing updated meta-analyses.

## Figures and Tables

**Table 1.** Characteristics of the Studies Included in the Current Systematic Review and Meta-analysis

Author	Year	Country	Sample (n)	PE (n)	NE (n)	Gender (M/F)	Methods	Stage	Grade (Low/High)	Treatment	Follw-Up (Months)	Sample Type
Stojnev, S.	2019	Serbia	404	276	128	312/92	IHC	I-II	81/194	TURBT ± mitomycin, Intravesical BCG, Cystectomy, Chemo/radiotherapy	25 to 125	Tissue
Shariat	2001	USA	51	19	32	47/4	ELISA	I-IV	7/44	Radical cystectomy and pelvic lymphadenectomy	45/7	Serum
Kim	2001	USA	80	51	29	65/15	IHC	I-IV	20/60	Radical cystectomy and pelvic lymphadenectomy	101/1	Tissue

BCG, Bacille Calmette-Guérin; ELISA, Enzyme-linked immunosorbent assay; IHC, Immunohistochemistry; NE, Negative expression; PE, Positive expression; TURBT, Transurethral removal of bladder tumor.

## References

[1] Patel VG Oh WK Galsky MD . Treatment of muscle‐invasive and advanced bladder cancer in 2020. CA Cancer J Clin. 2020;70(5):404 423. (10.3322/caac.21631) 32767764

[2] Richters A Aben KKH Kiemeney LALM . The global burden of urinary bladder cancer: an update. World J Urol. 2020;38(8):1895 1904. (10.1007/s00345-019-02984-4) 31676912 PMC7363726

[3] Ghasemi H Mousavibahar SH Hashemnia M Karimi J Khodadadi I Tavilani H . Transitional cell carcinoma matrix stiffness regulates the osteopontin and YAP expression in recurrent patients. Mol Biol Rep. 2021;48(5):4253 4262. (10.1007/s11033-021-06440-8) 34086159

[4] Sylvester RJ Rodríguez O Hernández V , et al. European Association of Urology (EAU) prognostic factor risk groups for non–muscle-invasive bladder cancer (NMIBC) incorporating the WHO 2004/2016 and WHO 1973 classification systems for grade: an update from the EAU NMIBC Guidelines Panel. Eur Urol. 2021;79(4):480 488. (10.1016/j.eururo.2020.12.033) 33419683

[5] Khansary S Tavilani H Ghasemi H . Gender, bladder cancer healthcare and burden of COVID-19. Cancer Investig. 2023;41(1):58 69. (10.1080/07357907.2022.2140351) 36282109

[6] Minoli M Kiener M Thalmann GN Kruithof-de Julio M Seiler R . Evolution of urothelial bladder cancer in the context of molecular classifications. Int J Mol Sci. 2020;21(16):5670. (10.3390/ijms21165670) 32784716 PMC7461199

[7] Zhu S Yu W Yang X Wu C Cheng F . Traditional classification and novel subtyping systems for bladder cancer. Front Oncol. 2020;10:102. (10.3389/fonc.2020.00102) 32117752 PMC7025453

[8] Gilyazova I Enikeeva K Rafikova G , et al. Epigenetic and immunological features of bladder cancer. Int J Mol Sci. 2023;24(12):9854. (10.3390/ijms24129854) 37373000 PMC10298356

[9] Galsky MD Balar AV Black PC , et al. Society for Immunotherapy of Cancer (SITC) clinical practice guideline on immunotherapy for the treatment of urothelial cancer. J Immunother Cancer. 2021;9(7). (10.1136/jitc-2021-002552) PMC8286774 34266883

[10] Su Y Feng W Shi J Chen L Huang J Lin T . circRIP2 accelerates bladder cancer progression via miR-1305/Tgf-β2/smad3 pathway. Mol Cancer. 2020;19(1):23. (10.1186/s12943-019-1129-5) 32019579 PMC6998850

[11] Meulmeester E Ten Dijke P . The dynamic roles of TGF‐β in cancer. J Pathol. 2011;223(2):205 218. (10.1002/path.2785) 20957627

[12] Ping Q Wang C Cheng X , et al. TGF-β1 dominates stromal fibroblast-mediated EMT via the FAP/VCAN axis in bladder cancer cells. J Transl Med. 2023;21(1):475. (10.1186/s12967-023-04303-3) 37461061 PMC10351189

[13] Zhang H Yue X Chen Z , et al. Define cancer-associated fibroblasts (CAFs) in the tumor microenvironment: new opportunities in cancer immunotherapy and advances in clinical trials. Mol Cancer. 2023;22(1):159. (10.1186/s12943-023-01860-5) 37784082 PMC10544417

[14] Shi X Yang J Deng S , et al. TGF-β signaling in the tumor metabolic microenvironment and targeted therapies. J Hematol Oncol. 2022;15(1):135. (10.1186/s13045-022-01349-6) 36115986 PMC9482317

[15] Kim JH Shariat SF Kim IY , et al. Predictive value of expression of transforming growth factor‐β1 and its receptors in transitional cell carcinoma of the urinary bladder. Cancer. 2001;92(6):1475 1483. (10.1002/1097-0142(20010915)92:6<1475::aid-cncr1472>3.0.co;2-x) 11745225

[16] MaruYama T Chen W Shibata H . TGF-β and cancer immunotherapy. Biol Pharm Bull. 2022;45(2):155 161. (10.1248/bpb.b21-00966) 35110501

[17] Fan Y Shen B Tan M , et al. TGF-β–induced upregulation of malat1 promotes bladder cancer metastasis by associating with suz12. Clin Cancer Res. 2014;20(6):1531 1541. (10.1158/1078-0432.CCR-13-1455) 24449823

[18] Zhang X-L Hu L-P Yang Q , et al. CTHRC1 promotes liver metastasis by reshaping infiltrated macrophages through physical interactions with TGF-β receptors in colorectal cancer. Oncogene. 2021;40(23):3959 3973. (10.1038/s41388-021-01827-0) 33986509

[19] Yu Y Wang W Lu W Chen W Shang A . Inhibin β-A (INHBA) induces epithelial–mesenchymal transition and accelerates the motility of breast cancer cells by activating the TGF-β signaling pathway. Bioengineered. 2021;12(1):4681 4696. (10.1080/21655979.2021.1957754) 34346300 PMC8806747

[20] Gholamin M Moaven O Memar B , et al. Overexpression and interactions of interleukin-10, transforming growth factor β, and vascular endothelial growth factor in esophageal squamous cell carcinoma. World J Surg. 2009;33(7):1439 1445. (10.1007/s00268-009-0070-y) 19440651

[21] Baek JY Morris SM Campbell J Fausto N Yeh MM Grady WM . TGF‐β inactivation and TGF‐α overexpression cooperate in an in vivo mouse model to induce hepatocellular carcinoma that recapitulates molecular features of human liver cancer. Int J Cancer. 2010;127(5):1060 1071. (10.1002/ijc.25127) 20020490 PMC2897914

[22] Naef M Ishiwata T Friess H Büchler MW Gold LI Korc M . Differential localization of transforming growth factor‐β isoforms in human gastric mucosa and overexpression in gastric carcinoma. Int J Cancer. 1997;71(2):131 137. (10.1002/(sici)1097-0215(19970410)71:2<131::aid-ijc1>3.0.co;2-1) 9139831

[23] Wang L Tong X Zhou Z , et al. Circular RNA hsa_circ_0008305 (circPTK2) inhibits TGF-β-induced epithelial-mesenchymal transition and metastasis by controlling TIF1γ in non-small cell lung cancer. Mol Cancer. 2018;17:1 18. 30261900 10.1186/s12943-018-0889-7 PMC6161470

[24] Ebert MP Fei G Schandl L , et al. Reduced PTEN expression in the pancreas overexpressing transforming growth factor-beta 1. Br J Cancer. 2002;86(2):257 262. (10.1038/sj.bjc.6600031) 11870516 PMC2375189

[25] Xiao W Wang X Wang T Xing J . Overexpression of BMP1 reflects poor prognosis in clear cell renal cell carcinoma. Cancer Gene Ther. 2020;27(5):330 340. (10.1038/s41417-019-0107-9) 31155610 PMC7237353

[26] Haque S Morris JC . Transforming growth factor-β: A therapeutic target for cancer. Hum Vaccin Immunother. 2017;13(8):1741 1750. (10.1080/21645515.2017.1327107) 28575585 PMC5557219

[27] Baz HGE Kamel MM Hammam OA Baz AGE . Potentials of transforming growth factors alpha and beta-1 in predicting the clinical outcome of bladder carcinoma. Int J Immunol Stud. 2010;1(2):169 182. (10.1504/IJIS.2010.034900)

[28] Ewart-Toland A Chan JM Yuan J Balmain A Ma J . A gain of function TGFB1 polymorphism may be associated with late stage prostate cancer. Cancer Epidemiol Biomarkers Prev. 2004;13(5):759 764. (10.1158/1055-9965.759.13.5) 15159307

[29] Chung JY-F Chan MK-K Li JS-F , et al. TGF-β signaling: from tissue fibrosis to tumor microenvironment. Int J Mol Sci. 2021;22(14):7575. (10.3390/ijms22147575) 34299192 PMC8303588

[30] Lenis AT Lec PM Chamie K Mshs MD . Bladder cancer: a review. JAMA. 2020;324(19):1980 1991. (10.1001/jama.2020.17598). 33201207

[31] Lee Y-C Lam H-M Rosser C Theodorescu D Parks WC Chan KS . The dynamic roles of the bladder tumour microenvironment. Nat Rev Urol. 2022;19(9):515 533. (10.1038/s41585-022-00608-y) 35764795 PMC10112172

[32] Shariat SF Shalev M Menesses-Diaz A , et al. Preoperative plasma levels of transforming growth factor beta1 (TGF-β1) strongly predict progression in patients undergoing radical prostatectomy. J Clin Oncol. 2001;19(11):2856 2864. (10.1200/JCO.2001.19.11.2856) 11387358

[33] Stojnev S Krstić M Čukuranović Kokoris J , et al. Prognostic impact of canonical TGF-β signaling in urothelial bladder cancer. Medicina (Kaunas). 2019;55(6):302. (10.3390/medicina55060302) 31238579 PMC6630377

[34] Hung T-T Wang H Kingsley EA Risbridger GP Russell PJ . Molecular profiling of bladder cancer: involvement of the TGF-β pathway in bladder cancer progression. Cancer Lett. 2008;265(1):27 38. (10.1016/j.canlet.2008.02.034) 18477502

[35] Zou J Huang R Li H , et al. Secreted TGF-beta-induced protein promotes aggressive progression in bladder cancer cells. Cancer Manag Res. 2019;11:6995 7006. (10.2147/CMAR.S208984) 31440088 PMC6664251

[36] Alshaker HA Matalka KZ . IFN-γ, IL-17 and TGF-β involvement in shaping the tumor microenvironment: the significance of modulating such cytokines in treating malignant solid tumors. Cancer Cell Int. 2011;11(1):33. (10.1186/1475-2867-11-33) 21943203 PMC3195702

[37] Giannelli G Villa E Lahn M . Transforming growth factor-β as a therapeutic target in hepatocellular carcinoma. Cancer Res. 2014;74(7):1890 1894. (10.1158/0008-5472.CAN-14-0243) 24638984

[38] Ashrafizadeh M Najafi M Orouei S , et al. Resveratrol modulates transforming growth factor-beta (TGF-β) signaling pathway for disease therapy: a new insight into its pharmacological activities. Biomedicines. 2020;8(8):261. (10.3390/biomedicines8080261) 32752069 PMC7460084

[39] Zhu H Chen H Wang J Zhou L Liu S . Collagen stiffness promoted non-muscle-invasive bladder cancer progression to muscle-invasive bladder cancer. Onco Targets Ther. 2019;12:3441 3457. (10.2147/OTT.S194568) 31123405 PMC6511250

[40] Kang HW Kim W-J Yun SJ . The role of the tumor microenvironment in bladder cancer development and progression. Transl Cancer Res. 2017;6(4);S758:744S.

[41] Xiong Y Zhang J Shi L , et al. NOGO-B promotes EMT in lung fibrosis via MMP14 mediates free TGF-beta1 formation. Oncotarget. 2017;8(41):71024 71037. (10.18632/oncotarget.20297) 29050340 PMC5642615

[42] Yang L Zhang F Wang X , et al. A FASN-TGF-β1-FASN regulatory loop contributes to high EMT/metastatic potential of cisplatin-resistant non-small cell lung cancer. Oncotarget. 2016;7(34):55543 55554. (10.18632/oncotarget.10837) 27765901 PMC5342435

[43] McConkey DJ Choi W Marquis L , et al. Role of epithelial-to-mesenchymal transition (EMT) in drug sensitivity and metastasis in bladder cancer. Cancer Metastasis Rev. 2009;28(3-4):335 344. (10.1007/s10555-009-9194-7) 20012924 PMC5915353

[44] Landry NM Dixon IMC . Fibroblast mechanosensing, SKI and Hippo signaling and the cardiac fibroblast phenotype: looking beyond TGF-β. Cell Signal. 2020;76:109802. (10.1016/j.cellsig.2020.109802) 33017619

[45] Fujii M Toyoda T Nakanishi H , et al. TGF-β synergizes with defects in the Hippo pathway to stimulate human malignant mesothelioma growth. J Exp Med. 2012;209(3):479 494. (10.1084/jem.20111653) 22329991 PMC3302232

